# Decreased Superoxide Dismutase Concentrations (SOD) in Plasma and CSF and Increased Circulating Total Antioxidant Capacity (TAC) Are Associated with Unfavorable Neurological Outcome after Aneurysmal Subarachnoid Hemorrhage

**DOI:** 10.3390/jcm10061188

**Published:** 2021-03-12

**Authors:** Harald Krenzlin, Dominik Wesp, Jan Schmitt, Christina Frenz, Elena Kurz, Julia Masomi-Bornwasser, Johannes Lotz, Florian Ringel, Thomas Kerz, Naureen Keric

**Affiliations:** 1 Department of Neurosurgery, University Medical Center Mainz, Langenbeckstr.1, 55131 Mainz, Germany; schmitt.j93@gmx.de (J.S.); christinafrenz@web.de (C.F.); elena.kurz@unimedizin-mainz.de (E.K.); junama@gmx.de (J.M.-B.); florian.ringel@unimedizin-mainz.de (F.R.); thomas.kerz@unimedizin-mainz.de (T.K.); naureen.keric@unimedizin-mainz.de (N.K.); 2 Institute of Clinical Chemistry and Laboratory Medicine, University Medical Center Mainz, Langenbeckstr.1, 55131 Mainz, Germany; johannes.lotz@unimedizin-mainz.de

**Keywords:** SAH, CSF, plasma, oxidative stress, ROS, antioxidant capacity, SOD

## Abstract

Background: Subarachnoid hemorrhage (SAH) is a devastating disease with high morbidity and mortality. Hypoxia-induced changes and hemoglobin accumulation within the subarachnoid space are thought to lead to oxidative stress, early brain injury, and delayed vasospasm. This study aimed to evaluate the antioxidant status and its impact on neurological outcome in patients with aneurysmal SAH. Methods: In this prospective observational study, 29 patients with aneurysmal SAH were included (mean age 54.7 ± 12.4). Blood and cerebrospinal fluid (CSF) samples were collected on days (d) 1, 3, and 7. In addition, 29 patients without intracranial hemorrhage served as controls. The antioxidant system was analyzed by glutathione peroxidase (GSH-Px; U/L) and total and free glutathione-sulfhydryl (GSH; mg/L) in the plasma. Superoxide dismutase (SOD, U/mL) and total antioxidant capacity (TAC, µmol/L) were measured in the serum and CSF. Clinical data were compiled on admission (Hunt and Hess grade, Fisher grade, and GCS). Neurological and cognitive outcome (modified Rankin scale (mRS), Glasgow Outcome Scale Extended (GOSE) and Montreal Cognitive Assessment (MoCA)) was assessed after 6 weeks (6 w) and 6 months (6 m). Results: Plasma levels of SOD increased from day 1 to 7 after SAH (d1: 1.22 ± 0.36 U/L; d3: 1.25 ± 0.33 U/L, *p* = 0.99; d7: 1.52 ± 0.4 U/L, *p* = 0.019) and were significantly higher compared to controls (1.11 ± 0.27 U/L) at day 7 (*p* < 0.001). Concordantly, CSF levels of SOD increased from day 1 to 7 after SAH (d1: 1.22 ± 0.41 U/L; d3: 1.77 ± 0.73 U/L, *p* = 0.10; d7: 2.37 ± 1.29 U/L, *p* < 0.0001) without becoming significantly different compared to controls (1.74 ± 0.8 U/L, *p* = 0.09). Mean plasma TAC at day 1 (d1: 77.87 ± 49.72 µmol/L) was not statistically different compared to controls (46.74 ± 32.42 µmol/L, *p* = 0.25). TAC remained unchanged from day 1 to 7 (d3: 92.64 ± 68.58 µmol/L, *p* = 0.86; d7: 74.07 ± 54.95 µmol/L, *p* = 0.8) in plasma. TAC in CSF steeply declined from day 1 to 7 in patients with SAH becoming significantly different from controls at days 3 and 7 (d3: 177.3 ± 108.7 µmol/L, *p* = 0.0046; d7: 85.35 ± 103.9 µmol/L, *p* < 0.0001). Decreased SOD levels in plasma and CSF are associated with a worse neurological outcome 6 weeks (mRS: CSF *p* = 0.0001; plasma *p* = 0.027/GOSE: CSF *p* = 0.001; plasma *p* = 0.001) and 6 months (mRS: CSF *p* = 0.001; plasma *p* = 0.09/GOSE: CSF *p* = 0.001; plasma *p* = 0.001) after SAH. Increased plasma TAC correlated with a worse neurological outcome 6 weeks (mRS: *p* = 0.001/GOSE *p* = 0.001) and 6 months (mRS *p* = 0.001/GOSE *p* = 0.001) after SAH. Conclusion: In our study, a reduction in the antioxidative enzyme SOD and elevated TAC were associated with a poorer neurological outcome reflected by mRS and GOSE at 6 weeks and 6 months after SAH. A lower initial SOD CSF concentration was associated with the late deterioration of cognitive ability. These findings support the mounting evidence of the role of oxidative stress in early brain injury formation and unfavorable outcome after SAH.

## 1. Introduction

Spontaneous subarachnoid hemorrhage (SAH), a devastating form of hemorrhagic stroke, accounts for 5–7% of all strokes and is associated with an exceptionally high disease-specific burden [1,2]. In total, 27% of stroke-related years of life lost are caused by SAH [3]. Initial mortality ranges from 15 to 20%, and one third die within one month [4,5]. The majority of spontaneous SAH—around 85%—are caused by the non-traumatic rupture of cerebral aneurysms (aSAH) [6]. Survivors of aSAH commonly face severe disability, and cognitive impairments such as deficits in memory, executive function, and language [7]. These long-term effects cause a high socio-economic burden [6]. The outcome depends on several factors, with the severity of the initial hemorrhage, the occurrence of delayed cerebral ischemia (DCI), and the patient age being among the most critical aspects [5]. In addition to the primary treatment, the occlusion of the source of bleeding [5], supportive treatments deal with preventing DCI and delayed cerebral vasospasms (DCV) occurring in 30% of the patients between days 4 and 10 [8]. However, DCI and angiographic vessel narrowing may occur independently and do not necessarily influence each other. The underlying pathomechanisms causing DCI and DCV include numerous different processes, including endothelial damage, the release of spasmogenic substances generated during the lysis of subarachnoid blood, changes in vascular responsiveness, inflammation, and immunological reactions of the vascular wall [9,10]. Despite significant changes in the management and treatment of SAH-associated complications over the last two decades, evidence of their impact on the improvement of morbidity and mortality remains scarce [5].

Recently, early brain injury (EBI) mechanisms have been strongly linked to morbidity and mortality after aSAH [11]. EBI comprises complex pathophysiological changes within the first 72 h after bleeding, including oxidative stress [11]. Oxidative stress describes an imbalance between reactive oxygen species (ROS) production and the antioxidant defense mechanism, leading to a situation of potential risk [12]. ROS consist of free radicals (e.g., superoxide anion, hydroxyl radical, peroxyl, and alkoxyl radical) and species that are able to generate radicals in situ (hydrogen peroxide, singlet oxygen, hypohalous acids). These may damage membrane lipids and DNA, and affect the function of cellular proteins [13]. The disruption of neuronal metabolism due to hypoxia after SAH causes an imbalance between ROS production and the intrinsic antioxidant system’s ability to prevent oxidative damage [14,15]. It is believed that these changes lead to the disruption of the blood–brain barrier (BBB) and subsequent edema, as well as vasoconstriction and the induction of apoptotic pathways such as p53, caspase-3, and caspase-9 [11,14,16]. Oxidative stress, in the wake of SAH, leads to altered levels of vasodilators (NO) and vasoconstrictors (endothelin 1) and consequent microvascular dysfunction and cerebral vasospasms [17]. During normal cellular respiration, superoxide dismutase (SOD), glutathione (GSH), and glutathione peroxidase (GSH-Px) are among the most important enzymatic scavengers [18]. As a first-line antioxidative defense, SODs are located ubiquitously in every subcellular compartment [19]. These systems become downregulated and overwhelmed after SAH, leading to an impaired antioxidative capacity [20]. Due to its high metabolic demand, the central nervous system is susceptible to ROS injury [21]. Because of their short half-life, the direct measurement of free radical levels in biological fluids is difficult [21]. It became common practice to measure plasma total antioxidant capacity (TAC) as the overall measure of cumulative antioxidant status instead of individual antioxidants [22].

This study aims to elucidate the interrelation of TAC, SOD, GSH, and GSH-Px measured in plasma and cerebrospinal fluid (CSF) with the neurological and cognitive outcome in patients with SAH.

## 2. Materials and Methods

Patient population: From February 2017 to February 2019, 29 consecutive patients (19 female and 10 male) who required an extra-ventricular drainage after aSAH were included in our study. The patients’ age ranged from 35 to 89 years (55 ± 12.4 years). An extra ventricular drain was placed on admission to our neurosurgical intensive care unit. Patients were followed-up until six months after discharge or until death occurred. CSF and clinical data were collected and analyzed prospectively. All patients received a thorough clinical examination on admission and before discharge from our hospital. Computed tomography (CT) was used to analyze hemorrhage characteristics and calculate the Fisher score. Neuromonitoring included continuous ICP measurement and transcranial doppler ultrasound. The clinical data and baseline characteristics are summarized in Table 1. Each patient’s clinical status at admission was graded according to the Hunt and Hess and Glasgow Coma Scale (GCS). The neurological outcome was assessed using the modified Rankin Scale (mRS) and Glasgow Outcome Scale Extended (GOSE), and the cognitive outcome using the Montreal Cognitive Assessment (MOCA) screening tool at 6 weeks and 6 months after SAH. In addition, 29 patients (57.5 ± 15.8 years, 15 female and 14 male) without intracranial hemorrhage served as controls. Vasospasm was defined as the deterioration in level of consciousness or development of new focal neurological signs related to ischemia that might be attributable to vasospasm and corresponding arterial narrowing on digital subtraction angiography after other possible causes of worsening were excluded.

Sampling procedure: CSF was obtained through external ventricular drainage on days 1, 3, and 7 after SAH. Concordant blood samples were obtained via an arterial cannula. The CSF of controls was obtained from either lumbar drainage placed for normal pressure hydrocephalus evaluation or the intraoperative opening of CSF spaces in meningioma and schwannoma patients. Samples were collected in a sterile plastic tube. The tube was centrifuged at 1500× *g* at 4 °C for 5 min. The supernatant was frozen, and aliquots were stored at −80 °C until analysis.

Measurement of analytes in the blood and CSF: TAC was measured in the plasma and CSF using a decolorization technique, where the radical cation 2,2′–azinobis-(3-ethylbenzothiazoline-6-sulfonic acid) was generated by reaction with potassium persulfate just before reaction with the plasma sample (ImAnOx^®^ (TAS/TAC) Kit, KC5200, Immundiagnostik AG, Germany). SOD (Superoxide Dismutase Assay Kit (No. 706002) Cayman Chemical, Ann Arbor, MI, USA) and GSH-Px (Glutathione Peroxidase Kit (No. RS 504), Randox Laboratories Ltd., Crumlin, UK) were measured via photometry. GSH was measured using high-performance liquid chromatography (HPLC).

Statistical analysis: Findings were reported as mean or median ± SD. For statistical analysis, we used the non-parametric Mann–Whitney U-test. Relations between TAC, SOD, GSH-Px, and clinical outcome parameters were analyzed using two-way analysis of variance (ANOVA) with Bonferroni‘s multiple comparison post hoc test using GraphPad Prism version 8.4.2 for macOS, GraphPad Software, La Jolla, CA, USA. A value of *p* < 0.05 was accepted as statistically significant.

## 3. Results

Clinical presentation: The median GCS on admission was 14, the median Hunt and Hess Classification (H&H) was 3, and the median Fisher score was 4. Of 29 patients, 13 developed symptomatic vasospasms. In six of these patients, DCI was confirmed. Six weeks after SAH, median mRS was 3 and median GOSE was 5. After 6 months, the median mRS was 1 and GOSE was 6. The median MOCA score 6 months after SAH was 28.

Plasma glutathione (GSH) and GSH-Px in patients with SAH and controls: No statistically significant differences were found in total and free GSH between patients with SAH (total GSH: 77.13 ± 27.77 mg/L, *p* = 0.95; free GSH: 67.13 ± 28.47 mg/L, *p* = 0.79) and controls (total GSH: 69.57 ± 25.82 mg/L, *p* = 0.95; free GSH: 61.34 ± 30.60 mg/L, *p* = 0.79). After SAH, no differences in the plasma levels of total GSH (d1: 69.57 ± 25.82 mg/L; d3: 69.33 ± 28.28 mg/L, *p* = 0.7228; d7: 72.55 ± 22.55 mg/L, *p* = 0.094) and free GSH (d1: 61.34 ± 30.60 mg/L; d3: 59.26 ± 27.93, *p* = 0.76; d7: 63.39 ± 24.06 mg/L, *p*= 0.97) were detectable between day 1 and days 3 or 7, respectively. Likewise, no statistically significant differences were found between GSH-Px levels measured at day 1, 3, and 7 in patients with SAH (d1: 2125 ± 534.8 U/L; d3: 1927 ± 592.4 U/L, *p* = 0.74; d7: 2000 ± 502.7 U/L, *p* = 0.95) and controls (2287 ± 690.5 U/L; *p* = 0.8395).

**Figure 1:**
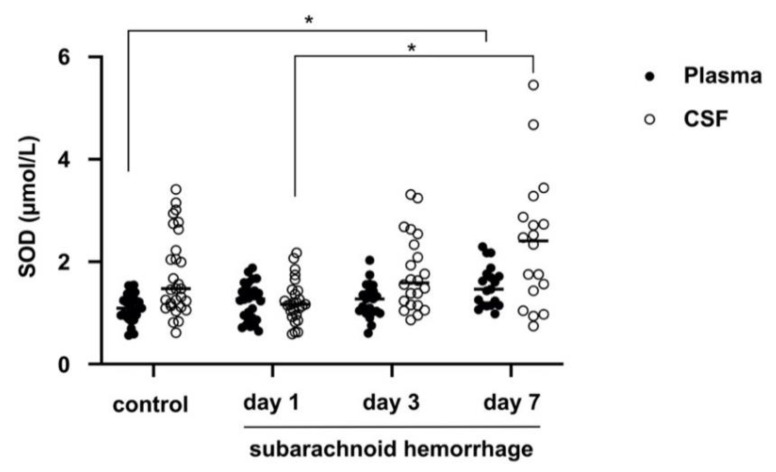
Superoxide dismutase (SOD) in plasma and cerebrospinal fluid (CSF) from patients with subarachnoid hemorrhage (SAH) and controls: Plasma and CSF levels of SOD increased from day 1 to 7 after SAH. *: *p* < 0.05.

SOD in plasma and CSF from patients with SAH and controls: Plasma levels of SOD increased from day 1 to 7 after SAH (d1: 1.22 ± 0.36 U/L; d3: 1.25 ± 0.33 U/L, *p* = 0.99; d7: 1.52 ± 0.4 U/L, *p* = 0.019) and were significantly higher compared to controls (1.11 ± 0.27) at day 7 (*p* < 0.001). Concordantly, CSF levels of SOD increased from day 1 to 7 after SAH (d1: 1.22 ± 0.41 U/L; d3: 1.77 ± 0.73 U/L, *p* = 0.10; d7: 2.37 ± 1.29 U/L, *p* < 0.0001) without becoming significantly different compared to controls (1.74 ± 0.8 U/L, *p* = 0.09) (Figure 1).

**Figure 2:**
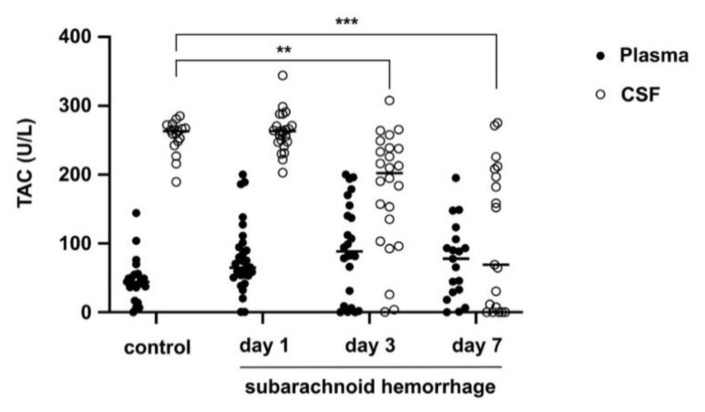
Total antioxidant capacity (TAC) in plasma and CSF from patients with SAH and controls. Mean plasma levels of TAC at day 1 were not statistically different compared to controls. TAC levels in CSF declined from day 1 to 7 after SAH becoming significantly different from controls at days 3 and 7 (*p* < 0.0001). **: *p* < 0.01; ***: *p* < 0.001.

TAC in plasma and CSF from patients with SAH and controls: Mean plasma levels of TAC at day 1 (d1: 77.87 ± 49.72 µmol/L) were not statistically different compared to controls (46.74 ± 32.42 µmol/L, *p* = 0.25). TAC plasma levels remained unchanged from day 1 to 7 (d3: 92.64 ± 68.58 µmol/L, *p* = 0.86; d7: 74.07 ± 54.95 µmol/L, *p* = 0.8). In similar fashion, CSF TAC levels were not different in patients with SAH (d1: 261.7 ± 29.15 µmol/L) compared to controls (255.0 ± 24.55 µmol/L, *p* = 0.99) at day 1. Nevertheless, TAC levels in CSF steeply declined from day 1 to 7 in patients with SAH becoming significantly different from controls at days 3 and 7 (d3: 177.3 ± 108.7 µmol/L, *p* = 0.0046; d7: 85.35 ± 103.9 µmol/L, *p* < 0.0001) (Figure 2).

**Figure 3:**
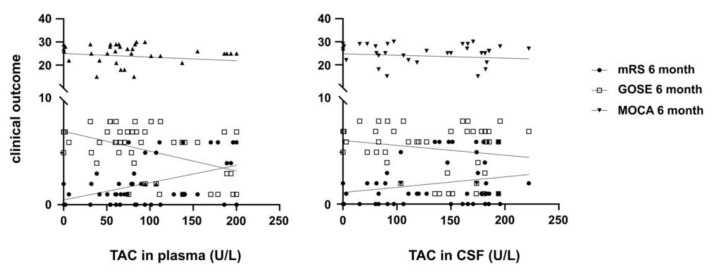
Correlation of TAC with clinical outcome. Increased plasma TAC correlated with a worse neurological outcome measured by modified Rankin Scale (mRS), Glasgow Outcome Scale Extended (GOSE) and MoCA after 6 weeks and 6 months (*p* = 0.011; *p* = 0.0007).

Correlation of TAC, SOD, and GSH/GSH-Px with clinical outcome: Decreased SOD levels in plasma and CSF are associated with a worse neurological outcome 6 weeks (mRS: CSF *p* = 0.0001; plasma *p* = 0.027/GOSE: CSF *p* = 0.001; plasma *p* = 0.001) and 6 months (mRS: CSF *p* = 0.001; plasma *p* = 0.09/GOSE: CSF *p* = 0.001; plasma *p* = 0.001) after SAH. As the only analyte, lower SOD levels in CSF were also related to cognitive decline 6 months after SAH (MoCA *p* = 0.001). The antioxidative capacity in CSF after SAH showed no significant correlation with the neurological (mRS and GOSE) or cognitive outcome (MoCA) at 6 weeks and 6 months after hemorrhage. In contrast, increased plasma TAC correlated with a worse neurological outcome 6 weeks (mRS: *p* = 0.001/GOSE *p* = 0.001) and 6 months (mRS *p* = 0.001/GOSE *p* = 0.001) after hemorrhage (Figure 3). The area under the curve (AUC) for TAC plasma concentration with mRS and GOSE after 6 months was 0.9734 (95% CI 0.9213 to 1.000, *p* < 0.0001) and 0.9615 (95% CI 0.8876 to 1.000, *p* < 0.0001) respectively.

**Figure 4:**
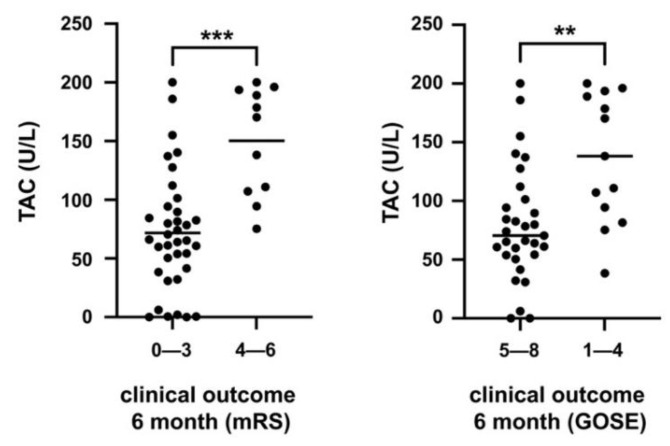
Unfavorable outcome 6 months after SAH is associated with increased plasma TAC levels (mRS: modified Rankin Scale, *p* = 0.0001; GOSE: Glasgow Outcome Scale Extended, *p* = 0.001). **: *p* < 0.01; ***: *p* < 0.001.

Likewise, an unfavorable outcome 6 months after SAH (mRS 4–6; GOSE 1–4) was statistically significantly associated with increased plasma TAC levels (mRS: *p* = 0.0001; GOSE: *p* = 0.001) (Figure 4).

Antioxidant capacity in CSF and plasma did not correlate with impaired cognitive outcome 6 weeks and 6 months after SAH. Poor clinical condition at the time of admission (H&H; GCS) and amount of subarachnoidal blood (Fisher scale) were not predictors of impaired TAC, but were individually associated with poor neurological outcome after 6 months (H&H: mRS *p* = 0.045, GOSE *p* = 0.035/Fisher scale: mRS *p* = 0.065, GOSE *p* = 0.12). In contrast, poor initial H&H and GCS correlated with higher SOD plasma levels (H&H: *p* < 0.0001; GCS: *p* < 0.0001). CSF and plasma TAC levels on day 1 did not correlate with symptomatic vasospasms, while their occurrence was associated with decreased CSF levels of TAC at day 7 (*p* = 0.049).

## 4. Discussion

In our study, we measured the anti-oxidative capacity in the plasma and CSF of patients with SAH. Lower concentrations of SOD in plasma and CSF were associated with an unfavorable neurological outcome at 6 weeks and 6 months after SAH. In contrast, high TAC levels in plasma were associated with a higher likelihood of a worse neurological outcome after SAH. TAC concentrations on day 1 were not related to the occurrence of vasospasms, while lower concentrations within the CSF were found in patients with symptomatic vasospasms at day 7. The measurements of GSH/GSH-Px were not indicative of an impaired prognosis.

Our data fall in line with previous articles addressing the importance of free radicals in EBI after SAH. Although there are many different sources that contribute to excessive free radical production, it is believed that mitochondrial overproduction and hemoglobin auto-oxidation are the most important [23]. The leakage of superoxide anions from mitochondria, caused by ischemic disruption of the electron transfer chain, accounts for most of the free radicals in EBI after SAH [18]. Ischemia in the wake of SAH causes Ca^2+^ accumulation in mitochondria [24], and the subsequent disruption of the mitochondrial membrane potential and the opening of membrane permeability transition pores [25]. Reestablishment of the membrane potential is achieved by increased substrate oxidation and O_2_ consumption, leading to the generation of superoxide [18]. The resulting excess of free radicals leads to an overload of the antioxidant systems, ultimately causing oxidative stress and the resulting damage to lipids, proteins, and DNA [18,23]. The delayed nature of this phenomenon might explain the time course of differences in TAC levels in the plasma and CSF of patients with SAH. It is speculated that free radical overload through mitochondrial breakdown leads to the depletion of antioxidant systems, oxidative stress, and ultimately neuronal cell death and detrimental neurological outcomes after SAH.

The occurrence of oxyhemoglobin within the subarachnoid space and CSF is another significant contributor of superoxide anions and hydrogen peroxide [26]. Ferryl hemoglobin, as another strong oxidant, is added through the interaction of metHb and oxyHb [26]. As these processes are confined to the subarachnoid space and CSF, they might explain the steep decline in TAC measured in the CSF from day 1 to 7 in our study. The effects caused by mitochondrial breakdown might be either too locally confined or too fast to be detected, such as the decline of plasma TAC levels. As effects resulting from cerebral ischemia are detected as early as 6 h after onset, the latter might be the case [27]. The chosen time point of day 1 in our study might represent a state in which changes to plasma TAC levels have already occurred, but changes to CSF TAC levels are beginning to unfold. The different sources of free radicals in the plasma and CSF might explain the differences observed in TAC levels in our study well. The association of lower circulating TAC levels with a more favorable outcome initially seems counterintuitive, but has been reported in many different diseases such as ischemic stroke, traumatic brain injury, and sepsis [28,29,30,31]. The pathophysiological processes underlying the reported findings are still not fully understood. Numerous results implicate higher sustained serum TAC levels in unfavorable outcomes in patients with septic shock. Elevated TAC levels might be a host response to severely propagating oxidative stress or a compensating mechanism for depleted antioxidative components. 

Serum uric acid (UA) is a free radical scavenger and is supposed to contribute to elevated serum TAC levels. UA has been independently correlated with mortality in septic patients. [32]. High UA levels are partially caused by renal failure in septic shock. Ischemia can convert xanthine dehydrogenase to xanthine oxidase, which in turn produces uric acid from purines [32,33,34]. Higher circulating TAC levels have been shown to coincide with increased lipid peroxidation, indicating higher oxidative stress [35]. Thus, higher levels of circulating TAC are considered to reflect a higher oxidative state than healthy controls. Ultimately, higher TAC levels have been proposed as predictive biomarkers for mortality in middle cerebral artery infarction patients [35]. Our study substantiates the concept of elevated circulating TAC levels as a predictor of an unfavorable outcome. So far, elevated circulating TAC has not been described in SAH, adding a novel aspect to the growing body of evidence. However, as the detailed mechanisms remain uncertain, future research to address these shortcomings is warranted.

SOD and GSH-Px are among the most essential enzymatic scavengers during physiological cellular respiration [18]. SOD produces H_2_O_2_ from O2^−^, which is scavenged by GSH-Px to prevent the accumulation of the more dangerous OH^−^ [36]. SAH leads to an increase in Cu-SOD and Zn-SOD activity and the SOD/GSH-Px ratio [20]. This change in balance between SOD and GSH-Px leads to the accumulation of OH^−^, subsequent oxidative stress, and consecutive EBI [23]. In our cohort, we observed an increase in SOD in the plasma and CSF of patients with SAH between days 1 and 7, while GSH-Px levels remained unchanged. These results fall in line with the observations made by Gaetani et al., who observed similar changes in 10 patients with SAH [37]. Lower concentrations of SOD, reflecting an impaired antioxidant defense, were associated with detrimental neurological outcomes in both studies [34]. A higher H&H grade and lower GCS on admission were associated with higher SOD levels. The resulting increase in the SOD/GSH-Px ratio reflects the changes described by Marzatico et al. and might add to oxidative stress after SAH via OH^−^ accumulation. In our study, the resulting increase in the SOD/GSH-Px ratio is not correlated with a detrimental neurological outcome. Still, it might contribute to impaired TAC and thus could add to neurological deficits after SAH.

Given the delayed nature of the changes leading to oxidative stress after SAH, it should come as no surprise that the initial GCS or Hunt and Hess Classification showed no correlation with decreased antioxidative capacity, but rather represent more independent predictors of an unfavorable outcome. However, higher Hunt and Hess grades correlated with increased SOD plasma levels. This increase might indicate SAH-related oxidative stress and the consequent increase in SOD activity and the SOD/GSH-Px ratio. During the time after SAH, this increase in oxidative stress might lead to an overpowering of the antioxidant defense mechanisms, which becomes evident in a declining TAC. This circumstance is highlighted by the fact that TAC concentrations within plasma and CSF at day one after hemorrhage were not associated with the occurrence of vasospasms during the curse of the disease. However, in patients with symptomatic vasospasm a significantly lower TAC was detected within the CSF at day 7. Although the occurrence of oxyhemoglobin within the subarachnoid space apparently contributes to oxidative stress, this is not reflected by the Fisher grade in our study. A possible explanation for this might be found in the lack of a more refined quantification of subarachnoid blood, rather than the small effects reflected within the antioxidant systems.

However, as the outcome after SAH is heterogenous and is influenced by a plethora of different factors, the rather small number of patients is a clear limitation of our study. The presented patient collective represents rather mild types of SAH with a median GCS of 14 and a more favorable outcome. It is speculated that more severe forms of SAH might lead to more differentiated results. Nevertheless, in the light of mounting evidence implicating oxidative stress in the pathophysiology of SAH induced brain injury, targeted therapies have become the focus of continued research efforts. So far, there are promising clinical and pre-clinical results of free radical scavengers such as docosahexaenoic acid, resveratrol, apigenin, paeoniflorin, and ebselen [38,39,40,41]. The lipid peroxidation inhibitor tirilazad and the superoxide scavenger PEG-SOD offer different alternatives to alleviate oxidative stress and potentially improve neurological outcome [42]. The optimal timing and dosage remain challenging to pinpoint as large-scale clinical trials are lacking. Therefore, further research, including larger prospective clinical trials, are warranted.

## 5. Conclusions

In our study, increased circulating TAC and decreased SOD plasma and CSF levels were associated with an unfavorable neurological outcome at both 6 weeks and 6 months after SAH. The initial H&H and GCS scores were correlated with higher levels of SOD in the plasma and CSF, indicating higher oxidative stress in patients with more severe bleeds. Furthermore, symptomatic vasospasm was associated with lower concentrations of TAC within the CSF 7 days after hemorrhage.

## Figures and Tables

**Table 1:** Baseline demographics and patient characteristics.

	ICH Patients	Control Subject
No. of subjects	29	29
Mean age (SD)	55.0 (12.4)	57.5 (15.23)
Sex		
Female	19	15
Male	10	14
Hunt and hess	3 (1.4)	n.a.
Fisher scale	4 (0.6)	n.a.
GCS	14 (5.3)	n.a.
WFNS	3 (1.7)	n.a.

ICH: intracerebral hemorrhage; SD: standard deviation; n.a.: not applicable; GCS: glasgow coma scale; WFNS: world federation of neurological surgeons.

## Data Availability

The data presented in this study are available on request from the corresponding author. The data are not publicly available due to restrictions of privacy and data security.
